# Chemotherapy during the last 30 days of life and the role of palliative care referral, a single center experience

**DOI:** 10.1186/s12904-022-00910-x

**Published:** 2022-02-07

**Authors:** Indryas Woldie, Tarek Elfiki, Swati Kulkarni, Colvin Springer, Eric McArthur, Nicole Freeman

**Affiliations:** 1 Windsor Regional Hospital, 2220 Kildare, WRCC, Windsor, ON N8W 2X3 Canada; 2 London Health Sciences Center, Windsor, Canada

**Keywords:** Chemotherapy, Palliative care, End of life

## Abstract

**Background:**

Chemotherapy use closer to the end of life is a marker of poor-quality care. There are now multiple studies and local reviews addressing this issue. Understanding the practice locally will give valuable insight and opportunity for improvement.

**Methods:**

The study is a retrospective chart review of patients on chemotherapy at the Windsor Regional Cancer Center who died between April 1^st^, 2016 to December 31^st^, 2018. Information on demographics, type of cancer, type, intent and route of chemotherapy, line of chemotherapy, referral to hospice and palliative care services was collected.

**Results:**

A total of 681 patients on chemotherapy died between April 1^st^, 2016 to Dec 13^th^, 2018. Of these, 119 (17.4 %) died within 30 days following chemotherapy. Chemotherapy was parenteral (Intravenous and Subcutaneous) for the majority (75.2%) of the patients. Most (66.4%) of the patients died of disease progression. Intent for chemotherapy was palliative in 85% of patients, adjuvant/neoadjuvant in 6.6% and curative in 8.4% of the patients. Chemotherapy was 1^st^, 2^nd^, 3^rd^ line or more in 67.4%, 21.3% and 11.3% of the patients respectively. The type of chemotherapy was conventional in 74.3% of patients and targeted/immunotherapy in 25.7% of patients.

Of the variables studied, lack of palliative referral and having lung cancer or melanoma were significantly associated with higher risk of getting chemotherapy within the last 30 days of life. The odds of getting chemotherapy within the last 30 days of life was 0.35, 95% CI (0.24-0.53), *P* <0.001 for those who were referred to palliative care. On the other hand, the odds of getting chemotherapy were 4.18, 95% CI (1.17-13.71), *P* = 0.037 and 2.21, 95% CI (1.24-4.01), *P* = 0.037 for those with melanoma and lung cancer respectively. In addition, those with early referral to palliative care (90 days or more prior to death) were least likely to receive chemotherapy within the last 30 days of life.

**Conclusion:**

Administration of chemotherapy within the last 30 days of life could cause unnecessary suffering to patients and cost to society. Early referral to palliative care was significantly associated with reduced risk of getting chemotherapy within the last 30 days of life in this study. Prospective study is recommended to further investigate the role of early palliative referral on use of chemotherapy during the last 30 days of life.

## Introduction

Chemotherapy use closer to the end of life is a marker of poor-quality care. It is a complex decision-making process that involves patient, family, oncologist and other members of the cancer care team. Chemotherapy at the end of life could decrease survival and increase health care utilization according to some studies [1–4].

Although the role of effective communication and involvement of palliative care has been emphasized as a solution, studies have shown considerable variation in transition from active therapy to end of life care [5]. A study from the United States showed many oncologists were reluctant to prescribe chemotherapy at the end of life. On the other hand, a patient’s decision depends on the clarity of information s/he receive [6].

A study from France showed patients who died in for profit hospitals, comprehensive cancer centers and centers without palliative care had greater than average use of chemotherapy near the end of life [7]. Very high rates of chemotherapy in the last 30 days of life were noticed in centers with no or limited palliative care services as well [8].

Another critical issue is the accuracy of oncologists’ predictions of survival. A study found out that oncologists made accurate predictions of survival in around a third of patients [9]. Finally, the role of chemotherapy in palliating symptoms should also be taken into account. This is especially true with the newer targeted and immunotherapeutic agents that are convenient to administer, could work quickly to palliate symptoms, and could be used more and more in the future [10].

This retrospective study will describe the rate of chemotherapy administered within the last 30 days of life and associated factors at a regional cancer center in Ontario, Canada. The study is unique as it gathered information on timing of palliative care referral both from the time of diagnosis as well as time of death and its relation to chemotherapy around the end of life.

## Methods

The study is a retrospective chart review of 681 patients on chemotherapy at the Windsor Regional Cancer Center who died between April 1^st^, 2016 and December 31^st^, 2018 per hospital records. Chemotherapy was defined as conventional/cytotoxic/, targeted therapy and immunotherapy based on the last chemotherapy regimen preceding patient’s death. Patients receiving hormonal therapy as the only cancer treatment were excluded. Information on demographics, type of cancer, type, intent and route of chemotherapy, line of chemotherapy and referral to palliative care services was collected. Additional information on time from diagnosis to palliative care referral as well as palliative care referral to death was also collected. Palliative care referral was considered early if patient was referred 90 days or more prior to death. These variables were selected during the inception of the study by the investigators based on their clinical judgment. Ethical clearance was obtained from the ethics review board of the Windsor Regional Hospital.

Continuous variables were presented as median (interquartile range) and categorical variables were presented as frequency (percentage). Univariable associations for factors of interest were estimated using odds ratios and their associated 95% confidence intervals, obtained from logistic regression models. Two-sided *p*-values <0.05 were considered statistically significant.

## Results

A total of 681 patients on chemotherapy died between April 1^st^, 2016 to Dec 31^st^, 2018. The median time for all patients from diagnosis to death was 1.73 years with an interquartile range of 0.82 to 3.46. Of these, 119 (17.4%) died within 30 days following chemotherapy, with a median of 17 days (range 1-30 days). Of those who died within 30 days of chemotherapy: 55.5% were male with a median age of 69 years. Intent for chemotherapy was palliative in 89.9% of patients, curative in 6.7% of the patients and adjuvant/neoadjuvant in 3.4%. Chemotherapy was 1^st^, 2^nd^, 3^rd^ line or more in 70.6%, 16% and 13.4% of the patients respectively. The type of chemotherapy was conventional in 73.9% of the patients and targeted/immunotherapy in 26.1 % of patients. Oral chemotherapy was used in a third (29.4%) of the patients, while the rest was parenteral (Intravenous or Subcutaneous). The three most common types of cancer in those patients who died within 30 days following chemotherapy were lung (30.3%), hematologic (22.7%) and gastro-intestinal (18.5%). Only 44.5% of the patients who died within 30 days of chemotherapy were referred to palliative care. Of these; 26%, 23.8% and 15.4% were referred within one year, 1-3 years and >3 years of diagnosis respectively. On the other hand, 38% of those referred to palliative care, were referred 90 days or more prior to death which is considered as early referral in this study (Table 1).

**Table 1:**
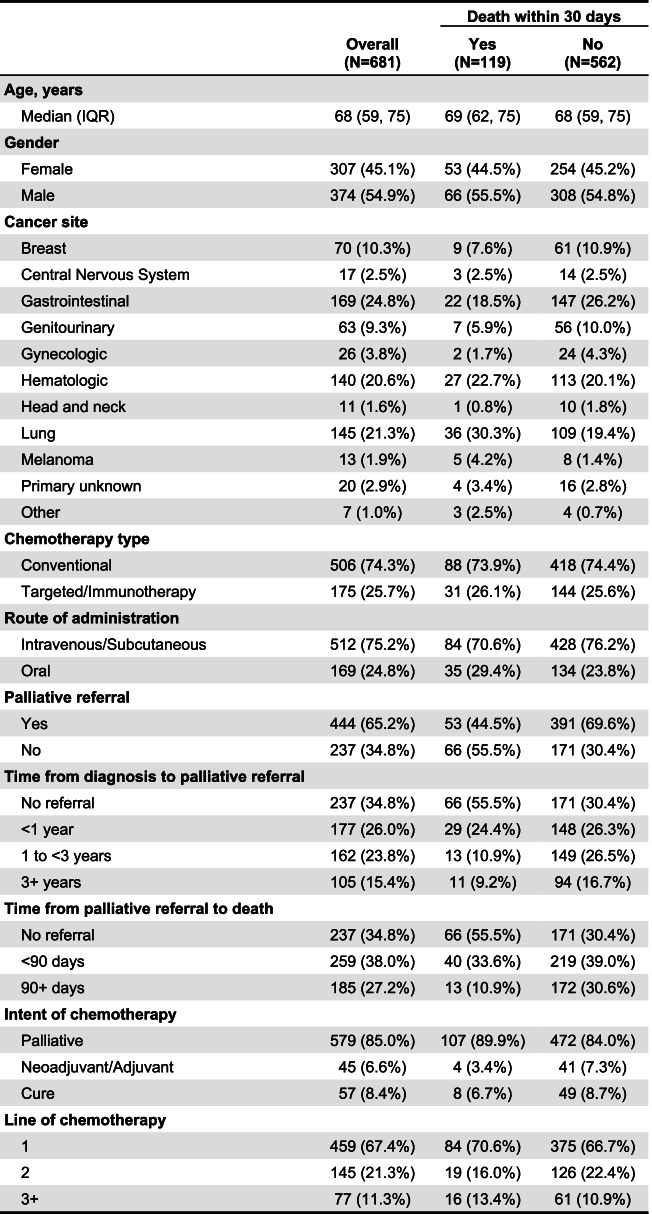
Baseline characteristics of study subjects

Almost half (49%) of the patients died in the hospital, whereas 15.5% passed away in hospice and 15.5% died at home. Place of death was unknown for 20 % of patients.

Of the variables evaluated, referral to palliative care and type of cancer were significantly associated with the likelihood of receiving chemotherapy during the last 30 days of life. Patients who were referred to palliative care were less likely to receive chemotherapy within the last 30 days of their life [OR 0.35, 95% CI (0.24-0.53), *P* <0.001]. In addition, those who had early referral to palliative care (referral 90 days or more prior to death) were least likely to receive chemotherapy within the last 30 days of life [0.2, 95% CI (0.1-0.36), *P* < 0.001]. On the other hand, patients who were referred to palliative care closest to diagnosis were more likely to have chemo within the last 30 days of life compared to those who were referred further from diagnosis Table 2.

**Table 2:**
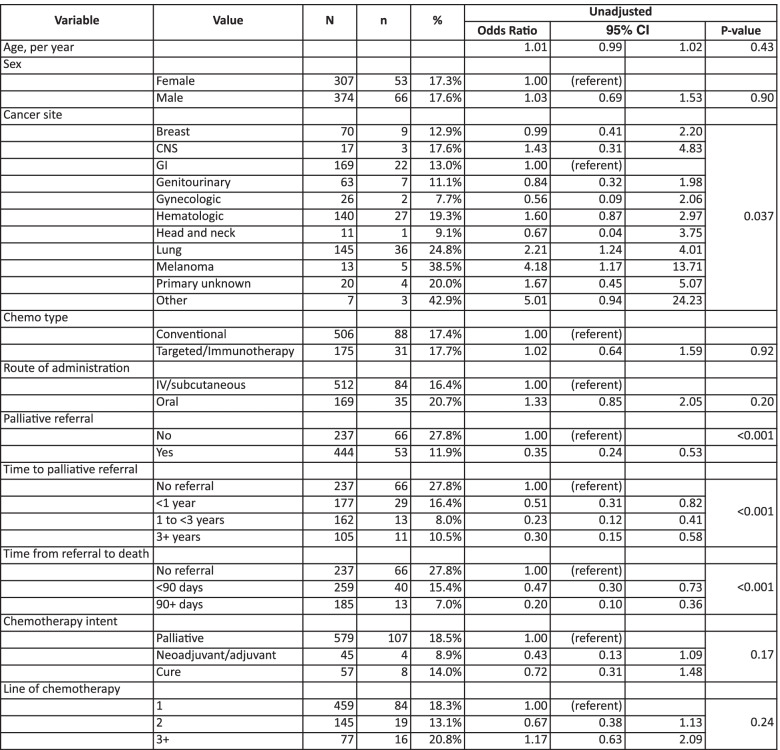
Association between study variables and odds of receiving chemotherapy within the last 30 days of life

With respect to cancer type, compared to gastrointestinal cancer, lung cancer [2.21, 95% CI (1.24-4.01), *P* = 0.037] and melanoma [4.18, 95% CI (1.17-13.71), *P* = 0.037] were significantly associated with higher odds of receiving chemotherapy within the last 30 days of life (Table 2).

## Discussion

The study showed 17.4 % of patients died within 30 days of chemotherapy which is similar to other studies from USA, France and Taiwan, but higher than a study from Switzerland that showed only 11.7% of patients received chemotherapy in the last 4 weeks of their life [7, 11–13].

However, our results are much lower than the data from Uganda and Egypt that showed 45% and 56% of patients in these countries, respectively, received chemotherapy in their last month of life [8, 14]. A possible explanation could be limited availability of palliative care services although cultural differences in end-of-life care could also play a role. Our study also showed patients who were referred to palliative care were less likely to receive chemotherapy within the last 30 days of their life. This is further supported by a study from France that showed young individuals, those treated in comprehensive cancer centers or high-volume centers without palliative care units were the most likely to receive chemotherapy near the end of life [7]. Another multicenter study from the USA showed quality of life near death is not improved and can be harmed by chemotherapy near death even in patients with good performance status [15, 16].

This is an interesting paradox particularly in low resource areas with limited palliative care service resulting in the use of more expensive and potentially harmful chemotherapy with futile outcome around the end of life. This suggests the need to build palliative care services hand in hand with active therapy in cancer treatment centers.

Our study showed only referral to palliative care and type of cancer were significantly associated with the likelihood of receiving chemotherapy in the last 30 days of life. Patients who are not referred to palliative care and those with either lung cancer or melanoma were more likely to receive chemotherapy within the last 30 days of their life. Further look into the association of palliative care referral and chemotherapy around the end of life showed those with early referral to palliative care (90 days or more prior to death), were least likely to receive chemotherapy within the last 30 days of life.

A study in Ontario showed younger age, male gender, hematologic malignancies and breast cancer were all associated with more aggressive care, which among others includes last dose of chemotherapy within the last 14 days of life [17]. Smaller sample size in our study might be the reason for the lack of statistically significant associations for some of the variables.

The benefits of early palliative referral cannot be overemphasized as there is evidence for improved quality of life and prolonged survival in patients referred to palliative care [18]. A study from New York showed chemotherapy in the last 30 days of life was associated with an increased rate of hospital admissions, emergency department visits, death in the hospital, fewer days in hospice care and a more than 50% increase in patient out of pocket costs for care. Another study from Taiwan showed similar findings [3, 15]. Our study also showed almost half of the patients who received chemotherapy within the last 30 days of life died in the hospital. This is partly explained by the fact that only 44% of the patients who recieved chemotherapy within the last 30 days of life were referred to palliative care.

The finding of more chemotherapy use in patients referred to palliative care closer to diagnosis in our study needs further investigation. One possible explanation could be earlier referral (from diagnosis) to palliative care for patients with more aggressive cancer who are likely to get at least first line palliative chemotherapy and die sooner). Further study, preferably prospective, incorporating ECOG performance status, reason for palliative referral and other relevant variables is recommended. We attempted to include ECOG performance status (PS) as a variable in our study, however information on PS was missing in most of the patients.

Although most studies around the use of chemotherapy closer to the end of life are retrospective, there are few prospective studies. A prospective study by Temel and colleagues showed patients receiving early palliative care who reported an accurate perception of their prognosis were less likely to receive intravenous chemotherapy near the end of life compared to those receiving standard of care (9.4% *v* 50%; *P* = .02) [19].

Finally, with the shift from conventional chemotherapy to targeted therapy with less toxicity and better efficacy is expected to change the landscape of cancer treatment making it more likely to see overlap between active therapy and palliative care [10]. However, this is not always the case particularly for those with low performance status as studies have shown no difference in survival among clinical trial ineligible patients (due to poor PS) between regular chemotherapy, chemotherapy and immunotherapy or immunotherapy alone. Another study actually showed significant toxicity from Immunotherapy [20, 21].

The study has several limitations including retrospective design; lack of details on circumstances of around the decision to stop chemotherapy which might be due to declining PS, Insurance coverage issue, patient/family decision among others; and relatively small sample size. Despite these limitations, it shed light on local practices with respect to chemotherapy use around end of life and associated factors.

## Conclusions

The use of chemotherapy in the last 30 days of life could result in inconvenience and unnecessary cost to patients and the health care system. Early palliative referral (90 days or more prior to death) is associated with less use of chemotherapy around the end of life in this study and is recommended. Further prospective studies and continuous audits on end-of-life care are also recommended.

## Data Availability

The datasets used and/or analyzed during the current study data/materials will not be shared publicly or deposited in a repository link due to institutional regulations, however will be available from the corresponding author on reasonable request.
